# A vulnerable journey towards professional empathy and moral courage

**DOI:** 10.1177/09697330221074013

**Published:** 2022-02-28

**Authors:** Anne Kari Tolo Heggestad, Anne-Sophie Konow-Lund, Bjørg Christiansen, Per Nortvedt

**Affiliations:** Faculty of Health Studies, 87368 VID Specialized University, Oslo and Centre for Medical Ethics, University of Oslo, Norway; Faculty of Health Studies, 87368 VID Specialized University, Vinderen, Oslo, Norway; Department of Nursing and Health Promotion, 60499 Oslo Metropolitan University, Oslo, Norway; Centre for Medical Ethics, University of Oslo, Oslo

**Keywords:** Empathy, moral courage, ethics education, nursing students, hidden curriculum

## Abstract

**Background:** Empathy and moral courage are important virtues in nursing and nursing ethics. Hence, it is of great importance that nursing students and nurses develop their ability to empathize and their willingness to demonstrate moral courage. **Research aim:** The aim of this article is to explore third-year undergraduate nursing students’ perceptions and experiences in developing empathy and moral courage. **Research design:** This study employed a longitudinal qualitative design based on individual interviews. **Participants and research context:** Seven undergraduate nursing students were interviewed during or immediately following their final clinical placement. **Ethical considerations:** The Norwegian Social Science Data Services (NSD) approved the study. Participants were informed that their participation was voluntary and were assured confidentiality. They were informed that they could withdraw from the study at any time, without providing reasons. **Findings:** Affective empathy seemed to be strong among third-year undergraduate nursing students. However, they tried to handle the situations in a ‘professional’ way, and to balance their emotions. At the same time, they expressed how difficult it can be to show moral courage when confronted with poor patient care. In addition, they spoke about a lack of role models during clinical practice and supervision. **Conclusions:** Undergraduate nursing students are in a vulnerable position throughout their journey to become professional and to develop empathy and moral courage. The professional socialisation and forming of professional empathy and moral courage among nursing students, may be seen as a complex interaction of formal and hidden curriculum, where role models play an important role. We argue that the main theme ‘Vulnerable students – a journey towards professional empathy and moral courage’ may cover the longitudinal project as a whole. This vulnerability is something both teachers and supervisors should be aware of when following up with students in their clinical placements.

## Introduction

Empathy and moral courage may be seen as two of the core virtues in nursing ethics and as fundamental concepts to ensure quality health care.^1,2^ Empathy may be defined as the ability to both cognitively and affectively understand and affirm another person’s feelings and experiences.^3^ Cognitive empathy is the capacity to deliberately take the perspective of another person, to try to understand his or her experiences, thoughts and feelings in particular circumstances, whereas affective empathy is the immediate and spontaneous identification with the feelings of that person.^4^ Empathy plays an essential role in relational communication between patients and health care workers;^5,6^ it is needed to understand – and thus provide proper care for – each patient’s needs.^7^ Research has shown that empathy may help to empower patients, influence patient satisfaction and even play an important role in healing.^8,9^ The ability to empathize is also associated with lower burnout among nurses and nursing students.^10,11^

In addition to empathy, nurses need moral courage when facing suffering or situations they find ethically blameworthy in their care provision. Moral courage may be understood as the ability to rise above fear, take action and stand up for one’s moral values, even if one risks negative consequences.^2,11^ It may also be seen as a part of nurses’ ethical competence.^12^ Moral courage as a concept has been debated in nursing since the era of Florence Nightingale.^13^

Prior research shows a loss of idealism and care among nursing students during education.^13^ Moreover, earlier research suggests that undergraduate nursing students experience a lack of moral courage, which may lead to moral distress.^1,14^ Konings et al. (2021) found that nursing students perceive themselves as courageous in situations where they interact directly with a patient. However, the authors also found that showing moral courage was more challenging when facing ethical dilemmas which involved other health professionals.^15^

The professional socialisation and forming of professional empathy and moral courage among nursing students, may be seen as a complex interaction of formal and hidden curriculum.^16,17^ In these interactions, both core values they have learned earlier in life, values in the formal curriculum and values in the hidden curriculum come into play. The formal curriculum is based on formal values and knowledge, like ethical theories and principles,^17^ while ‘hidden curriculum’ may be understood as the indirect learning of unwritten rules, implicit values, social behaviours and attitudes usually seen in clinical settings.^16–18^

Developing professional empathy and moral courage for nursing students is a relational process, which may be seen as a part of the professional socialisation. Clinical placement and role models’ actions and behaviours play a vital role in this socialisation and learning process.^16,17,19,20,21^ Student’s interactions and socialisations with role models, and the hidden curriculum they represent in clinical placement, may therefore be of great importance in developing professional empathy and courage.

## Research aim

The aim of this article is to describe and discuss undergraduate nursing students’ perceptions and experiences in developing professional empathy and moral courage. The article centres around the following research question:

• What challenges do undergraduate students experience with regards to developing professional empathy and moral courage during their clinical placement?

## Research design

This article is part of a qualitative, longitudinal study in which we followed undergraduate students from their first through their third year of nursing education. Here, the aim was to explore what characterizes undergraduate nursing students’ development of empathy.

Findings from interviews with the students in their first year have been published earlier.^22^

## Participants and research context

Colleagues of the researchers were asked to inform first-year nursing students about the project during the students’ first clinical placement. Those who agreed to participate were then provided with further information about the project and gave their written consent. We did not include students that we were supervising, since this could have had impacted their answers. The students who were interviewed in their first year were informed that they would be contacted for a follow-up study during their third year. Eleven students participated in the first study,^22^ seven of whom participated in the follow-up study in their third year. All seven – six women and one man – were interviewed. The participants ranged from 20 to 32 years of age; four were from Norway, one was from another Scandinavian country, and two were from Asia.

## Interviews

The interviews were conducted in a meeting room at the school, in a meeting room at the location of their clinical practice, or in one of the researchers’ offices. We explained to the students that the aim of the interview was to follow up findings from the first-year interviews. We used a thematic guide in the interviews, but tried to ask open-ended questions to let the students speak as freely as possible and not simply confirm our preunderstandings. In addition, we reminded them about what they had told us in the interviews in their first year.

## Analysis

We followed Braun and Clarke’s six steps of analysis, which include: (1) becoming familiar with the material by reading it openly, (2) generating tentative codes, (3) searching for themes, (4) evaluating themes, (5) defining and naming themes and (6) writing the text or article.^23^ The interviews were first read with an open mind, to try to get an overall view of the potential themes. The steps described above resulted in subthemes which could be abstracted to main themes. In this article, we present the main theme ‘Vulnerable students – a journey towards professional identity’, with the subthemes ‘Vulnerable students striking a professional balance’, ‘Vulnerable students and the importance of good role models’ and ‘Vulnerable students struggling with showing moral courage’.

The first author conducted the main analysis for this article. However, all the authors read through the interviews and discussed the interpretations and findings (Table 1).

**Table 1.** Abstraction of teams.

Quotations	Subthemes	Main theme
One should try to help, but not becoming too involved and too touched. But that is impossible, I won’t manage it, because then I will become cold. So it is a difficult balance, I think	Vulnerable students striking a professional balance	Vulnerable students - a journey towards professional empathy and moral courage
One of them, she was very realistic in a way, and very kind. And she was good. One could see that she had done this before, in a way, that she knew exactly what to say, and that sometimes you do not need to say much at all. (…) She seemed to be confident. (student describing a role model)	Vulnerable students and the importance of good role models’	
It has to be serious, if I’m going to mention it to my nurse-supervisor in practice, as a student. If I was one of the staff, I think I would be more straightforward, but not as a student. (…) It is the role of a student’	Vulnerable students struggling with showing moral courage’	

## Ethical considerations

Norwegian Social Science Data Services (NSD) approved the study. The participants who signed an informed consent form were told that they could withdraw from the study at any time, without consequences, and without providing reasons.

## Findings

The affective aspect of empathy appeared strong among the third-year undergraduate nursing students, as was also found in the first study.^22^ However, in this follow-up study, students also spoke about how to handle the situations in a ‘professional’ way, and how they now managed to balance their affective and cognitive empathy. At the same time, they also talked about the challenges they experienced in their journey to become professionals, and how difficult it could be to show moral courage when confronted with patient suffering and poor patient care.

### Vulnerable students striking a professional balance

All the students talked about being emotionally affected, especially in situations where patients were suffering, and in situations where they observed poor patient care. At the same time, they were concerned about how to handle their feelings and emotions in a professional way, and the importance of being professional and developing professional empathy in these situations. In their first year, they spoke about not knowing how to handle all their emotions – however, now, they detailed the strategies they used to balance their emotions and not become too affected by the situation. As one of the students (Student A) said, ‘You’ve got to learn to handle it, and you should behave professionally. You should not sit down with the patient and be sad’.

Becoming professional was experienced as a journey in which they tried to find the right balance between distance and emotions in the situation: they wanted to show that they cared for the patients, without being overwhelmed by emotions. One student (Student C) explained: ‘If they sit there crying, I can’t just start crying and sobbing (…). But I have to show that I care, if you know what I mean. It’s something in between’.

Finding this balance was not easy, however, and some described it as almost impossible. If they were ‘too professional’, they were afraid of becoming ‘cold’. It seemed that they experienced a kind of ambivalence regarding how to handle their emotions. This may be interpreted as balancing on the edge – or a fine balance – as described in the following quotation: ‘One should try to help, but not become too involved and too affected. But that is impossible. I won’t manage it, because then I will become cold. So, it is a difficult balance, I think’ (Student D).

Another strategy the students used to be – or become – professional, was to try to find distance: not only from the situation, but also towards the patient involved in the situation. The students tried to keep in mind that they were occupying a role in which they represented a profession: in other words, that they were more than just their personal selves in that moment. As this student stated, ‘You have to think that you have a role. When I am at work as a nurse, (…) I do represent a profession and I am more me as a professional than I am me as a person’ (Student D).

Some of the students related that they often did not feel much of anything in the moment, though they sometimes had a reaction once they went home. However, this lack of emotion sometimes concerned them; in these instances, they sought advice from their supervisors, parents or friends. As this student explained:

I felt like a horrible person who didn’t feel anything when I was in the midst of it. But I felt a lot when I got home. So I talked to my supervisor and mom and dad and such, and they said that maybe that is what being professional is. That you can be present in the moment, because it isn’t my role to stand and cry, even if it is awful. But that I sort of feel it when I get home. (Student E)

### Vulnerable students and the importance of good role models

Several of the nursing students described how they experienced the ability or inability of their professional role models – the nurses or doctors with whom they collaborated – to empathize with patients. When they encountered health care professionals who did seem to be affected by their patients’ suffering, this was something the students appreciated: ‘I think it is good to see those who have been nurses for a long time, that they still may be affected’ (Student E). However, the students also described how some of the health care professionals seemed emotionally ‘cold’, while the students reported that they were still often strongly affected emotionally when patients were suffering ‘Well, I can tell when they [the doctors] are through medical school, to put it that way – that they have put away their feelings. I think so. At least many of them, I think, have put away their feelings’ (Student E).

The students also seemed to be surprised about how some nurses and doctors spoke about seriously ill patients and death and dying.

I think about the attitudes of some of the nurses—I heard one say, ‘Ugh! I wish he would die tonight so I don’t have to sit by the death bed’. It’s one of those practical things you think about. You can think it, but not say it to your colleagues. (Student B)

The students also pointed to some nurses’ and doctors’ problematic lack of empathy in their encounters with patients. As one student explained, ‘The people [health care workers] are good, but maybe – what should I say – their empathy has faded away. They forget to put themselves in the situation of another person, to see how he is’ (Student F).

However, the students also spoke about differences they saw between nurses and doctors: they found doctors to be less empathic than the nurses, and felt some nurses had an important role in empathizing with the patients when doctors lacked this ability. The students described some doctors as ‘terrible in their communication with the patients’, and some who seemed to feel that communicating with patients was not really their job.

He [the doctor] had been washing his hands, with his back to the patient, and informed the patient that ‘your cancer has come back.’ And the patient was very upset, of course. (…) I mean, he never looked the patient in the eye. (…) I don’t know why he doesn’t show any kind of emotion, or care for his patient in this situation, but he turned to the nursing student, with a look that said ‘Now…this is your job’. (Student D)

Others talked about nurses who seemed to be empathic and confident when communicating with patients in difficult situations:

One of them, she was very realistic in a way, and very kind. And she was good. One could see that she had done this before, in a way, that she knew exactly what to say, and that sometimes you do not need to say much at all. (…) She seemed to be confident. (Student G)

### Vulnerable students struggling with showing moral courage

Some of the situations the students described were those which the nursing students thought were ethically problematic. One of the students also thought the nurses were being cowardly in not confronting doctors about their poor behaviour. Although the students wanted to react when they observed bad behaviour and poor practice, they did not want to confront the nurses or doctors themselves. They expressed concern that a confrontation would lead to consequences. Indeed, when they were in the role of student, they thought a situation needed to be ‘seriously bad’ to justify a reaction: ‘It has to be serious, if I’m going to mention it to my nurse-supervisor in practice, as a student. If I was one of the staff, I think I would be more straightforward, but not as a student. (…) It is the role of a student’ (Student C).

The students offered several reasons for not speaking up on behalf of the patients. Some were afraid of how they would be evaluated at the end of their clinical placement if they criticized or questioned the nurses or doctors’ behaviour. They were also afraid of negatively impacting their employment options once they had finished their clinical placement and hence jeopardizing their career. One of the students described how she experienced being on the lowest stage of the professional hierarchy. She thought it would be easier to speak up when entering into the role as a registered nurse.

Instead of speaking up, most of them tried to make the best of and accept the situation.

You get, in a way…you just accept how the situation is. And then you try to make the best of it. Because I do not see any other opportunities now (…). I think that many of us think that we want to make a difference. Everybody wants to make a difference, but when you are in the midst of the situation, it requires some guts, and a will, in a way. (Student D)

Although the students were afraid of speaking up when encountering what they felt were unethical situations, some of them also spoke about situations where they did show personal engagement and managed to stand up for the patients. One of the students spoke about how angry and provoked she became when the nurses did not let a patient speak to a doctor. Though she thought it was extremely challenging, and she felt like it was a ‘rebellion’, she managed to persuade her supervisor to call the doctor. This same student stated that she did not want to work in a profession of ‘cowards’.

## Discussion

The professional socialization of nursing students in clinical placement is a complex process, where both formal and ‘hidden curriculum’ may come into play. Our findings from interviews with third-year nursing students show that undergraduate nursing students may experience their role as vulnerable. Within this vulnerability and complexity, they are trying to develop professional empathy, balance their emotions while finding the right distance towards patients. However, this development of professionality seems to be lonely, with few good role models, and the students experience it as challenging to stand up in the face of patient suffering and unethical situations. Although the main theme – ‘Vulnerable students on their moral journey’ – was identified when analysing the interviews with the third-year students, this theme arguably represents their entire journey of developing professional empathy and moral courage, from students’ first through third year of nursing education. This highlights the importance of maintaining awareness of nursing students’ vulnerability throughout their education.

### A striking balance towards moral professionality

According to earlier research, clinical placement is of great importance when learning to become professional.^21,24^ However, our findings show that entering the professional world as novices may be challenging. The third-year nursing students in our study talked about how they struggled, but also how they coped with situations in which they saw patients suffering.

When these students were interviewed about the same topics in their first year, the students were often overwhelmed by their emotions and did not know how to handle them. They thought that being emotionally affected was important aspect of empathy, and expressed that they were afraid of losing this ability.^22^ By their third year, however, they seemed to have learned different strategies to handle the situations, which they described as a way of being more professional. These strategies may be seen as a result of a professional socialization in clinic, where the hidden curriculum may play an essential role.^17^

One of these strategies was to try to maintain emotional distance in certain situations. Similar strategies are found among medical students, when they describe what it means to be professional.^25^ To be sure, maintaining emotional distance in some situations may be a good strategy, if one is still able to care for one’s patients and meet their needs. If this leads to a decline of empathy, however, it may be a threat to patient care. Here, it seems that the nursing students must maintain a fine balance, and how they are followed up by role models may be crucial for their learning process and hence development of professional empathy.^17,26^ As we argue in one of the previous articles from this longitudinal study, being professional does not mean putting empathy aside, but learning how to handle one’s emotions.^22^

### The importance of role models

Both nurses and doctors are important role models for undergraduate nursing students trying to find the right professional balance and handling ethical challenging situations; as such, they have a moral responsibility in how they meet and treat vulnerable patients who are suffering.^26^ They also have a responsibility to guide students in ethically proper behaviour. However, earlier research among nurses in hospitals, show that nurses struggle with how to handle ethical challenges themselves, which may lead to experiences of emotional ‘immunization’.^27^ This represents a problematic challenge, however, when nursing students encounter role models they describe as ‘cold’ and without sensitivity towards suffering patients. In an article on earlier findings from this study, we reported that the first-year nursing students also described nurses and doctors as cynical, and they expressed a fear of becoming cold and indifferent themselves.^22^

There is a common belief that most nurses and doctors fulfil their responsibilities to patients and act with moral character and great care and competency. Nevertheless, and as our findings show, some do fail to act responsibly – we know this from earlier research on empathy in medical school and from unethical behaviour reported in elder care and nursing homes.^25,27–29^ Recently, a larger cross-sectional explorative study on elder abuse in Norwegian nursing homes showed that, out of 3693 nursing staff (response rate 60.1%), 76% had observed one or more incidents of elder abuse during the past year, and 60.3% reported they had perpetrated one or more incidents of abuse in the same period. Psychological abuse and neglect were most commonly reported.^30^ This indicates that nurses as well as physicians occasionally fail in their responsibilities as good role models for nursing students, and that the students experience a mismatch between the ideals and formal curriculum taught in school, and ‘the hidden curriculum’ in practice. Even more problematic is that patients are being treated badly and basic ethical codes are being neglected, as are legal frameworks and patient rights. Such instances of ethically problematic behaviour should be taken more seriously by faculty, hospital staff and administrators. According to earlier research, negative role models may be one of the major barriers to professional identity formation among students.^31,32^ This suggests that those responsible for clinical placement for undergraduate students should be aware of who is likely to best supervise students; moreover, supervisors should be given the opportunity to take courses on how to supervise effectively.

### Lack of moral courage among students and their role models

According to Bjarnason and Lasala, moral courage is a ‘sacred’ value in nursing.^33^ However, our findings show that many nursing students may not have developed moral courage, and that they often fail to speak up against nurses or doctors perceived as violating patient rights or causing them harm. Moreover, they are not encouraged to speak out, and there seems to be no system through which students can anonymously report unethical behaviour. Finally, there do not appear to be people or support systems to whom they can turn in such times of crises and moral stress.

Paradoxically, students may experience serious consequences if they fail in their care or display unethical behaviour, while the threshold for professionals to encounter sanctions seems much higher. The fact that both nurses and nursing students fail to speak up for patients and may lack moral courage is well known from previous research.^1,14,34–37^ Registered nurses might fail to speak up because they are afraid of losing their job or they may fear negative reactions from colleagues.^2^ One reason why students do not speak up, may be that they are afraid of the consequences they may face if they report bad behaviour, fearing reprimands or threats to employment;^1,38^ this was also a concern among the students we interviewed in our study. Another reason may be students’ desire to be accepted,^1^ and that they do not want to jeopardize their study progress. As our findings show, they may also lack confidence in their relationship with their supervisor or teacher from school because of hierarchical issues. If nurses – who are among the most important role models for nursing students – do not speak up for the patients, it is even more difficult for nursing students to do so.

Research indicates that not speaking up when confronted with morally inadequate situations may lead to moral distress among nurses and nursing students,^36,39,40,41^ which in turn may lead to burnout and a desire to leave the profession.^42^ Building moral courage during nursing students’ education may therefore be of great importance.

### Recommendations for educators and clinical preceptors.

Educational institutions have a moral responsibility to follow up on nursing students’ development of professional empathy, and to educate nurses to have the moral courage necessary for ethical practice. Indeed, earlier studies show that students need support to develop moral courage.^41^ Research indicates that learning ethics may be important when building moral courage.^14^ One way to support students in this could be by thematizing empathy and moral courage when students are in clinical placement, by letting the students discuss and debrief on situations they experience as ethical challenging.^17,43^ Rudolph et al. (2006) argue for the ‘debriefing with good judgement’ approach, which focuses on how to share critical messages in a safe context. Another way could be to let students participate in ethical discussions, such as in ethics reflection groups, and hence increase their competency in resolving ethical dilemmas.^36^ Research shows that ethics reflection groups in clinical practice may help nursing students cope with ethical challenges, thus increasing their ethics competency and improving their moral attitude.^44,45^ Ethics reflection and debriefing on ethical challenging situations, may also help students and their mentors be aware of the hidden curriculum their learning process, and how this may affect the professional development.

According to previous research, it may also be important to build mutually respectful relationships between mentors and students, to make students more confident.^1^

Teaching nursing students how to care for themselves may also help them avoid burnout and cynicism. Here, strategies could be to teach them mindfulness techniques, self-reflection and emotional skills.^46^ Further, and importantly, nursing education should support students in registering concerns about unethical practices, and should discuss and validate their experiences, as well as the facts related to each case. Moreover, education should provide personal support to all students experiencing difficult and stressful situations.

### Strengths and limitations

One limitation is that we did not have the opportunity to interview all the students who participated in the study in their first year of nursing education.

## Conclusions

Our research identified several failures and challenges regarding the teaching of third-year nursing students, particularly related to their aim to become good and ethically responsible care providers. The students’ moral character should be nurtured towards their becoming proficient nurses who are ready to enter a range of positions in health care. Instead, we see that the students encounter improper behaviour and a lack of empathy from their role models. Although the students oppose unethical practices, react to cynical behaviour and strive to be empathetic and caring, they lack support in becoming morally courageous, nor is there an institutional system through which they can (anonymously) voice their concerns. Here, we see great potential for educational improvement around moral development, which teachers and nursing schools should cultivate and strengthen.
